# Synthesis and *In Vitro* Protein Tyrosine Kinase Inhibitory Activity of Furan-2-yl(phenyl)methanone Derivatives

**DOI:** 10.3390/molecules16064897

**Published:** 2011-06-14

**Authors:** Fei Lang Zheng, Shu Rong Ban, Xiu E Feng, Cheng Xiao Zhao, Wenhan Lin, Qing Shan Li

**Affiliations:** 1 School of Pharmaceutical Science, Shanxi Medical University, Taiyuan 030001, Shanxi, China; 2 State Key Laboratory of Natural and Biomimetic Drugs, Peking University, Beijing 100083, China

**Keywords:** halophenols, furan-2-yl(phenyl)methanone, protein tyrosine kinases inhibitor, structure-activity relationships (SAR)

## Abstract

A series of novel furan-2-yl(phenyl)methanone derivatives were synthesized, and their structures were established on the basis of ^1^H-NMR, ^13^C-NMR and mass spectral data. All the prepared compounds were screened for their *in vitro* protein tyrosine kinase inhibitory activity and several new derivatives exhibited promising activity, which, in some cases, was identical to, or even better than that of genistein, a positive reference compound. The preliminary structure-activity relationships of these compounds were investigated and are discussed.

## 1. Introduction

Bromophenols isolated from various marine algae, ascidians and sponges have recently attracted much attention due to their unique structures and varied pharmacological activities, which include antioxidative [1], protein tyrosine kinase (PTK) inhibitory [2], anticancer [3], protein tyrosine phosphatase 1B inhibitory [2], antithrombotic [4], antimicrobial [5], anti-inflammatory [6], enzyme inhibitory [7], cytotoxic [8] and appetite suppressant [9] effects. The core structures of these bromophenols are two benzene rings connected by a methylene or carbonyl group. However, studies on their structure optimization and their corresponding structure-activity relationship (SAR) with PTK inhibitors have been rarely reported, despite the fact that several natural bromophenol compounds [10] and some new derivatives with antimicrobial activities were prepared [5,11].

Protein kinases play an important role as cell function regulators in signal transduction pathways that regulate a number of cellular functions, such as proliferation, growth, differentiation, death and various regulatory mechanisms. A variety of tumor types have dysfunctional growth factor receptor tyrosine kinases, which result in inappropriate mitogenic signaling. PTKs are, therefore, attractive targets in the search for therapeutic agents, not only against cancer, but also in many other diseases [12,13].

A wide range of heterocyclic ring systems has been studied for the development of novel chemical entities as lead molecules in drug discovery. Introduction of appropriate heterocycles into a lead compound is a common strategy during the drug discovery process. It has been found that many common rings, including different heterocyclic and simplified aromatic structures, are important as PTK inhibitors [14]. The encouraging activities of our previously prepared bromophenols [15] prompted us to investigate new analogs involving further modification of five-membered heterocyclic rings, to optimize the SAR that might lead to potent and selective biological activity. The furan ring is a very important bioactive structure that is considered to be a basic building block in the design and synthesis of new drugs. Furan rings are electron-rich systems that are amenable to act as good ligands for metal ions. Furan derivatives that are substituted at the 2- and 5- positions are frequently found in nature. These derivatives show broad-spectrum pharmacological properties [16]. Hence, in this study, we modified the structures of bromophenols by replacement of one benzene ring with a furan ring. The introduction of new substituents and functional groups at various positions on aromatic or heteroaromatic fragments of a potential drug might lead to changes in its molecular shape that allows optimum binding to the receptor, as well as its physicochemical properties that affect drug distribution and metabolism [17,18]. The scaffold was designed in such a way that the benzene ring was connected with heteroaryl system by a carbonyl group, with the hope of attaining superior biological activity. A series of new furan-2-yl(phenyl) methanone derivatives was synthesized by convenient methods to evaluate their biological and PTK inhibitory activities. Finally, the preliminary SARs were investigated.

## 2. Results and Discussion

**Scheme 1:**
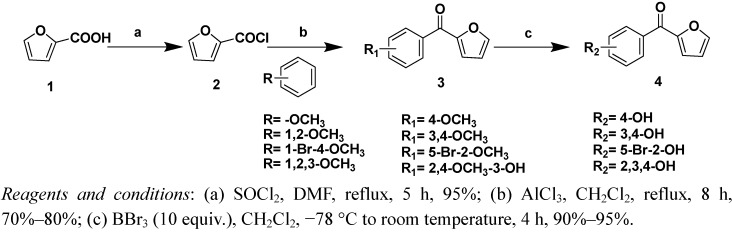
Syntheis of compounds 3a–3d and 4a–4d.

**Scheme 2:**
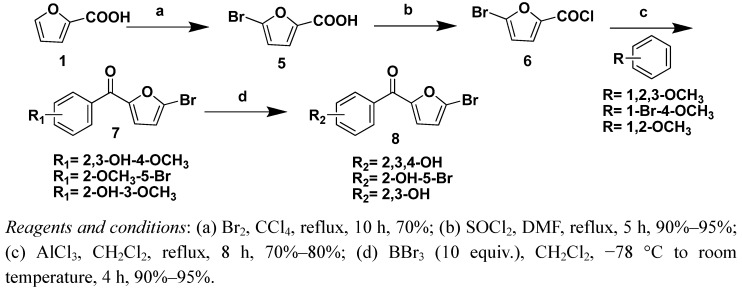
Syntheis of compounds 7a–7c and 8a–8c.

**Scheme 3:**
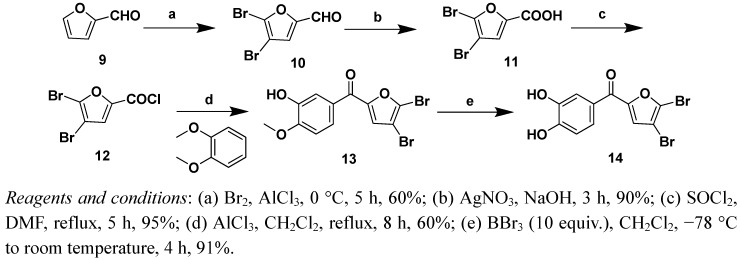
Syntheis of compounds 13 and 14.

**Scheme 4:**
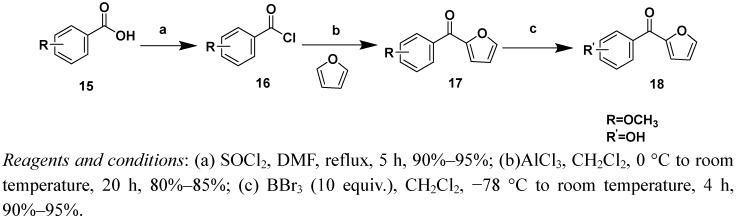
Syntheis of compounds 17a–17b and 18a–18b.

**Scheme 5:**
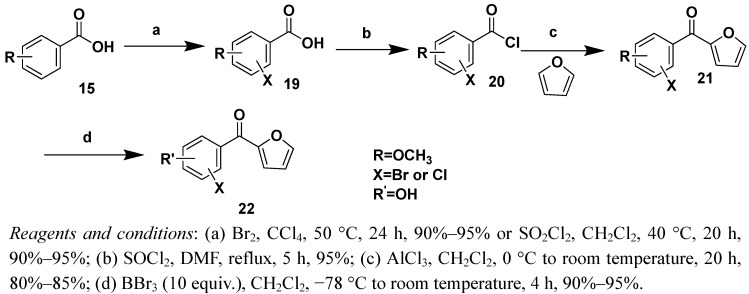
Syntheis of compounds 21a–21d and 21a–21d.

Two strategies were adopted to prepare the target compounds. One strategy was Friedel-Crafts acylation of substituted benzene derivatives with the furoyl chloride (Scheme 1, Scheme 2 and Scheme 3), and the other was Friedel-Crafts acylation of furan with substituted benzoyl chloride derivatives (Scheme 4 and Scheme 5).

The synthetic routes of compounds **3a–3d** and **4a–4d** are shown in Scheme 1. The starting material, furan-2-carboxylic acid, was reacted with dry SOCl_2_ in the presence of a catalytic amount of *N,N*-dimethylformamide (DMF) to yield furan-2-carbonyl chloride, which was reacted further with substituted benzene derivatives to yield compound **3**. Compound **4** was obtained by treating compound **3** with BBr_3_.

5-Bromofuran-2-carboxylic acid (**5**) and 4,5-dibromofuran-2-carboxylic acid (**11**) were prepared according to the previously described procedures [19,20], which were also used to prepare compounds **7a–7c**, **8a–8c**, **13** and **14** (Scheme 2 and Scheme 3).

**Figure 1:**
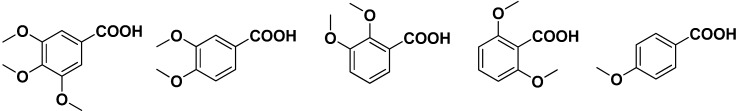
Structures of starting materials **15**.

The synthetic routes to compounds **17a**/**17b** and **18a**/**18b** are illustrated in Scheme 4. Variously substituted benzoic acids **15** (Figure 1) were refluxed in anhydrous SOCl_2 _to yield acyl chlorides **16**, which were reacted with furfural catalyzed by AlCl_3_. The product was purified by silica gel column chromatography (ligarine/EtOAc 80:20, v/v) to give compound **17**, which were treated with BBr_3_ to give compound **18**.

To examine the influence of the number and position of halogen atoms on their bioactivites, chlorination of **15** (Figure 1) with SO_2_Cl_2_ or bromination with Br_2_ provided a good yield of **19**. Chloro- and bromo-substituted compounds **21** and **22** were then synthesized according to the second route (Scheme 5). The structures of target compounds are shown in Table 1.

**Table 1:** Structures of the target new compounds. 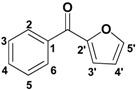

Compounds	Substituent Group	Compounds	Substituent Group
**3a**	3,4-OCH_3_	**4a**	3,4-OH
**3b**	2,4-OCH_3_, 3-OH	**4b**	2,3,4- OH
**3c**	2-OCH_3_, 5-Br	**4c**	2-OH, 5-Br
**3d**	4-OCH_3_	**4d**	4-OH
**7a**	2,3-OH, 4-OCH_3_, 5’-Br	**8a**	2,3,4-OH, 5’-Br
**7b**	2-OCH_3_, 5-Br, 5’-Br	**8b**	2-OH, 5-Br, 5’-Br
**7c**	3-OH, 4-OCH_3_, 5’-Br	**8c**	3,4-OH, 5’-Br
**13**	3-OH, 4-OCH_3_,4’,5’-Br	**14**	3,4-OH, 4’,5’-Br
**17a**	2,3-OCH_3_	**18a**	2,3-OH
**17b**	2,6-OCH_3_	**18b**	2,6-OH
**21a**	3-Br, 4-OCH_3_	**22a**	3-Br, 4-OH
**21b**	2,6-Br, 3,4,5-OCH_3_	**22b**	2,6-Br, 3,4,5-OH
**21c**	2-Cl, 3,4,5-OCH_3_	**22c**	2-Cl, 3,4,5-OH
**21d**	2-OH, 3-OCH_3_, 4,5-Br	**22d**	2,3-OH, 4,5-Br

### 2.1. Results

As shown in Table 2, all of the furan-2-yl(phenyl)methanone derivatives were subjected to the *in vitro* PTK inhibitory activity testing using the PTK assay, as previously reported [13]. Some of the new derivatives exhibited promising activity, which in some cases, was identical to, or even better than that of genistein, a positive reference compound in the same model. Thus, compounds **4a**, **4b**, **8a**, **8c** and **22c** exhibited PTK inhibitory activity (IC_50_ values 4.66, 6.42, 5.31, 2.72 and 4.62 μM, respectively), which was more potent than that of the genistein (IC_50_ 13.65 μM). Compounds **18a**, **18b** and **22d** also displayed moderate PTK inhibitory activity (IC_50_ values 13.23, 12.65 and 9.56 μM, respectively).

**Table 2:** PTK inhibitory activity.

Compounds	Protein Tyrosine Kinase (PTK) inhibition activity ^a^	Compounds		Protein Tyrosine Kinase (PTK) inhibition activity ^a^
**3a**	NA	**4a**		**4.66**
**3b**	NA	**4b**		**6.42**
**3c**	NA	**4c**		NA
**3d**	NA	**4d**		NA
**7a**	NA	**8a**		**5.31**
**7b**	NA	**8b**		NA
**7c**	NA	**8c**		**2.72**
**13**	NA	**14**		NA
**17a**	NA	**18a**		**13.23**
**17b**	NA	**18b**		**12.65**
**21a**	NA	**22a**		NA
**21b**	NA	**22b**		NA
**21c**	NA	**22c**		**4.62**
**21d**	NA	**22d**		**9.56**

^a^ IC_50_ (μM). Values are means of three experiments; NA, not active at 5 μg/mL. Genistein 13.65 μM.

### 2.2. SAR Analysis

The results indicated that the number of hydroxyl groups had a great effect on the PTK inhibitory activity. More than one hydroxyl group was essential for the activity, as none of the compounds that had only one hydroxyl group in the phenyl ring showed any activity, whereas those with more than one exhibited high to moderate activity, although the activities did not increase directly with the number of hydroxyl groups. For example, when three hydroxyl groups were present in the phenyl ring, the corresponding activity was lower than that with two hydroxyl groups. Among the hydroxyl substituted derivatives, the three compounds **4a**, **18a** and **18b** with a similar structure that had two hydroxyl groups in the benzene ring had strong to moderate activity. However, they did not show equal activity, which suggested that two substituent hydroxyl groups at appropriate positions in the benzene ring were important to the PTK inhibitory activity of these compounds. 

Comparison of the activities of the halogen-substituted derivatives indicated that the halogen atoms in the phenyl ring (**22b** and **22c**) contributed to the PTK inhibitory activity in the order of Cl > Br. However, compound **22b** with a fully-substituted phenyl ring showed no activity.

We can conclude that hydroxyl groups on the furan-2-yl(phenyl)methanone backbone are essential for the *in vitro* PTK inhibitory activity of these compounds, and introduction of a methoxyl group can lead to the disappearance of activity, as all the methoxyl derivatives were inactive. The presence of one or more halogen atoms on the phenyl ring also increases the activity. The number and position of the hydroxyl groups and halogen atoms in the phenyl ring could influence the activity potency.

By comparison of the activity of compounds **4a**, **4b**, **8a** and **8c**, we found that the introduction of a Br atom at the C-5 position in the furan ring could increase activity. However, compound **14** showed no activity due to the introduction of another Br atom at the C-4 position in the furan ring of **8c** which showed the strongest activity. The results suggest that a Br atom substituted at the C-5 position in the furan ring could enhance the PTK inhibitory activity, but Br atoms substituted at the C-4 position in are inhibitory for such activity.

## 3. Experimental

### 3.1. General

Melting points were determined on a XT4A microscopic stage melting point apparatus with an aromatic temperature control system. NMR spectra were recorded on a Bruker-AV400 spectrometer with TMS as an internal standard and DMSO-d6 or CDCl_3_ as the solvent. Chemical shifts (d values) and coupling constants (*J* values) were given in ppm and Hz, respectively. ESI mass spectra were obtained on an API QTRAP 3200 LC-MS spectrometer，the high-resolution mass spectra were obtained on a Bruker Daltonics Apex IV 70e FTICR-MS (Varian 7.0T). TLC analysis was carried out on silica gel plates GF254 (Qingdao Haiyang Chemical, China). Silica gel column chromatography was performed with silica gel 60 G (Qingdao Haiyang Chemical). Commercial solvents were used without any pretreatment, whereas dichloromethane was dried by refluxing and distilling over calcium hydride.

### 3.2. Chemistry

#### 3.2.1. Procedure for the synthesis of 5-bromofuran-2-carboxylic acid **5**

Br_2_ (8 mL) was slowly added to a solution of 2-furancarboxylic acid (**1**, 14.0 g) in CCl_4_ (60 mL). The reaction mixture was stirred at 45–50 °C for 24 h. The solvent was then removed under reduced pressure to yield a red solid which was recrystallized from boiling water to give the compound **5**.

#### 3.2.2. Procedure for the synthesis of 4,5-dibromofuran-2-carboxylic acid **11**

Freshly distilled furfural (10.0 g) was added dropwise with mechanical stirring at 0 °C to aluminum chloride (32.0 g) over a two hour period. Bromine (37.0 g) was then added dropwise at 0 °C over a two hours period, after which stirring was discontinued and the reaction mixture allowed to stand overnight. The reaction was quenched by carefully pouring the mixture into ice (800 mL) and then extracting the aqueous layer three times with ether. The combined organics were washed twice with saturated sodium bicarbonate, once with brine, and dried with anhydrous MgSO_4_. The solvent was removed under reduced pressure to yield a red oil. Purification using column chromatography on silica (hexanes/ethyl acetate V/V = 1:1 as eluent) yielded compound **10** (13.2 g, R_f_ = 0.45, 20% ethyl acetate in hexanes) as an orange oil. Next compound **10** (13.2 g), H_2_O (40.0 mL) and AgNO_3_ (8.0 g) were added into a flask and the mixture was stirred at room temperature for 4 h. Then the reaction was completed, 6 M hydrochloric acid adjusted to PH to 2~3, and extracting the aqueous layer three times with ethyl acetate. The combined organics were dried with Na_2_SO_4_. The solvent was removed under reduced pressure, the solid separated was recrystallized from boiling water to give compound **11**.

#### 3.2.3. General procedure for synthesis of compounds **3a–3d**, **7a–7c** and **13**

A solution of 2-furancarboxylic acid (**1**, 1.0 g) and a catalytic amount of DMF in thionyl chloride (5 mL) was stirred at 80–90 °C for 5 h. After concentration under reduced pressure, furoyl chloride **2** was dissolved in dried CH_2_Cl_2_ and reacted with 1,2-dimethoxybenzene (1 mL) catalyzed by AlCl_3_. The reaction mixture was heated to 50–60 °C for 12 h. After the reaction was completed, the reaction was quenched by carefully pouring the mixture into iced water (100 mL), extracting the aqueous layer three times with CH_2_Cl_2_, and drying with anhydrous Na_2_SO_4_. The solvent was removed under reduced pressure to yield a yellow oil. The residue was purified by silica gel column chromatography (petroleum ether/EtOAc 80:20, v/v) to give compound **3a** as a light yellow solid (1.66 g, 80% yield). 

*(3,4-Dimethoxyphenyl)(furan-2-yl)methanone* (**3a**). Mol. formula (MW): C_13_H_12_O_4_ (232 g/mol); mp: 98–100 °C; ^1^H-NMR (CDCl_3_) δ 7.72 (d, *J* = 8.4 Hz, 1H, Ph-6-H), 7.67(s, 1H, Ph-2-H), 7.55 (d, *J* = 1.6 Hz, 1H, 5’-H), 7.22 (d, *J* = 3.6 Hz, 1H, 3’-H), 6.92 (d, *J* = 8.4 Hz, 1H, Ph-5-H), 6.57 (d, *J* = 2.0 Hz, 1H, 4’-H), 3.94 (s, 6H, CH_3_); ^13^C-NMR (CDCl_3_) δ 56.2, 56.2, 110.1, 111.8, 112.0, 119.6, 124.1, 129.9, 146.6, 148.6, 152.6, 153.1, 181.0; ESI-MS (%): *m/z* = 233 (100) [M+H]^+^, 255 (100) [M+Na]; HRMS (ESI): Calcd. for [M+H]^+^: 233.0808; Found: 233.0811.

*Furan-2-yl(3-hydroxy-2,4-dimethoxyphenyl)methanone* (**3b**). Mol. formula (MW): C_13_H_12_O_5_ (248 g/mol); light yellow solid; Yield: 75%; mp: 168–170 °C; ^1^H-NMR (DMSO-d6) δ 8.13 (d, *J* = 9.2 Hz, 1H, Ph-6-H), 7.73 (d, *J* =1.6 Hz, 1H, 5’-H), 7.38 (d, *J* = 3.6 Hz, 1H, 3’-H), 6.64 (dd, *J* = 3.6 Hz, *J* = 1.6 Hz, 1H, 4’-H), 6.58 (d, *J* =9.2 Hz, 1H, Ph-5-H), 3.97, (s, 3H, OCH_3_), 3.93 (s, 3H, OCH_3_); ^13^C-NMR (DMSO-d6) δ 56.1, 60.7, 103.1, 103.2, 112.4, 120.3, 128.2, 136.6, 146.8, 152.2, 158.3, 158.5, 183.9; ESI-MS (%): *m/z* = 249 (100) [M+H]^+^; HRMS (ESI): Calcd. for [M+H]^+^: 249.0758; Found: 249.0759.

*(5-Bromo-2-methoxyphenyl)(furan-2-yl)methanone* (**3c**). Mol. formula (MW): C_12_H_9_BrO_3_ (281 g/mol); light yellow solid; Yield: 70%; mp: 66–70 °C; ^1^H-NMR (CDCl_3_) δ 7.68 (s, 1H, 5’-H), 7.59 (dd, *J* = 8.8 Hz, *J* = 2.8 Hz, 1H, Ph-4-H), 7.54 (d, *J* = 2.8 Hz, 1H, Ph-6-H), 7.09 (d, *J* = 4.0 Hz, 1H, 3’-H), 6.91 (d, *J* = 8.8 Hz, 1H, Ph-3-H), 6.58 (dd, *J* = 4.0 Hz, *J* = 2.0 Hz, 1H, 4’-H), 3.98 (s, 3H, OCH_3_); ^13^C-NMR (CDCl_3_) δ 56.1, 112.4, 112.5, 113.4, 120.8, 129.6 , 131.9, 134.7, 147.6, 152.5, 156.5, 181.2; ESI-MS (%): *m/z* = 281(100) 283 (98.7) [M+H]^+^ 303 (100) 305 (98.7%) [M+Na].

*Furan-2-yl(4-methoxyphenyl)methanone* (**3d**). Mol. formula (MW): C_12_H_10_O_3_ (202 g/mol); light yellow solid;Yield: 80%; mp: 58–60 °C; ^1^H-NMR (CDCl_3_) δ 8.06 (s, 1H, Ph-2-H), 8.04 (s, 1H, Ph-6-H), 7.70 (s, 1H, 5’-H), 7.25 (d, *J* = 3.6 Hz, 1H, 3’-H), 7.01 (s, 1H, Ph-3-H), 6.99 (s, 1H, Ph-5-H), 6.61 (d, *J* = 3.6 Hz, 1H, 4’-H), 3.90 (s, 3H, OCH_3_); ^13^C-NMR (CDCl_3_) δ 55.5, 112.1, 113.7, 114.1, 119.7, 129.8, 131.7, 132.2, 146.6, 152.7, 163.3, 181.2; ESI-MS (%): *m/z* = 203 (100) [M+H]^+^ 225 (100) [M+Na].

*(5-Bromofuran-2-yl)(2,3-dihydroxy-4-methoxyphenyl)methanone* (**7a**). Mol. formula (MW): C_12_H_9_BrO_5_ (313 g/mol); light yellow solid; Yield: 70%; mp: 128–130 °C; ^1^H-NMR (DMSO-d6) δ 11.01 (br s, 1H, OH), 8.83 (br s, 1H, OH), 7.43 (dd, *J* = 8.8 Hz, *J* =2.4 Hz, 1H, Ph-6-H), 7.38 (d, *J* = 3.6 Hz, 1H, 3’-H), 6.93 (d, *J* = 3.6 Hz, 1H, 4’-H), 6.71 (d, *J* = 8.8 Hz, 1H, Ph-5-H), 3.88 (s, 3H, OCH_3_);^ 13^C-NMR (DMSO-d6) δ 56.4, 104.2, 115.4, 122.4, 122.4, 123.3, 129.2, 134.5, 150.1, 153.3, 153.6, 182.0; ESI-MS (%): *m/z* = 313 (100) 315 (99.1) [M+H]^+^ 335 (100) 337 (99.1%) [M+Na]; HRMS (ESI): Calcd. for [M + H]^+^:312.9706; Found: 312.9708.

*(5-Bromo-2-methoxyphenyl)(5-bromofuran-2-yl)methanone* (**7b**). Mol. formula (MW): C_12_H_8_Br_2_O_3_ (360 g/mol); light yellow solid; Yield: 76%; mp: 98–100 °C; ^1^H-NMR (CDCl_3_) δ 7.59 (dd, 1H, *J* = 8.8 Hz, *J* = 2.4 Hz, Ph-4-H ), 7.53 (d, *J* = 2.4 Hz, 1H, Ph-6-H), 7.01 (d, *J* = 3.6 Hz, 1H, 3’-H ), 6.91 (d, *J* = 8.8 Hz, 1H, Ph-3-H ), 6.53 (d, *J* =3.6 Hz, 1H, 4’-H ), 3.81 (s, 3H, OCH_3 _); ^13^C-NMR (CDCl_3_) δ 56.1, 112.6, 113.6, 114.5, 122.5, 128.9, 129.9, 132.0, 135.1, 154.1, 156.6, 179.8; ESI-MS (%): *m/z* = 359 (51) 361(100) 363 (50) [M+H]^+^ 381 (51) 383 (100) 385 (50) [M+Na].

*(5-Bromofuran-2-yl)(3-hydroxy-4-methoxyphenyl)methanone*(**7c**). Mol. formula (MW): C_12_H_9_BrO_4_ (297 g/mol); light yellow solid; Yield: 72%; mp: 128–130 °C; ^1^H-NMR (CDCl_3_) δ 7.67 (s, 1H, Ph-2-H), 7.62 (d, *J* = 8.4 Hz, 1H, Ph-6-H), 7.32 (d, *J* = 3.6 Hz, 1H, 3’-H), 7.12 (d, *J* = 8.4 Hz, 1H, Ph-5-H), 6.50 (d, *J* = 3.6 Hz, 1H, 4’-H) 3.90 (s, 3H, OCH_3_); ^13^C-NMR (CDCl_3_) δ 56.2, 111.1, 113.8, 118.0, 119.6, 122.1, 123.4, 127.9, 148.6, 152.6, 155.1, 181.0; ESI-MS (%): *m/z* = 295 (100) 297 (98.9) [M−H]^−^; HRMS (ESI): Calcd. for [M+H]^+^:296.9757; Found: 296.9768.

*(4,5-Dibromofuran-2-yl)(3-hydroxy-4-methoxyphenyl)methanone* (**13**). Mol. formula (MW): C_12_H_8_Br_2_O_4_ (376 g/mol); light yellow solid; Yield: 60%; mp: 107–109 °C; ^1^H-NMR (CDCl_3_) δ 7.66 (d, *J* = 8.4 Hz, 1H, Ph-5-H), 7.54 (s, 1H, 3’-H), 7.23 (s, 1H, Ph-2-H), 7.02 (d, *J* = 8.4 Hz, 1H, Ph-6-H), 3.98 (s, 3H, OCH_3_); ^13^C-NMR (CDCl_3_) δ 56.2, 104.3, 111.5, 114.1, 123.2, 124.9, 128.4, 129.0, 146.8, 150.9, 153.6, 180.0; ESI-MS (%): *m/z* = 373 (51) 375 (100) 377 (50) [M−H]^−^; HRMS (ESI): Calcd. for [M+H]^+^: 374.8865; Found: 374.8867.

#### 3.2.4.General procedure for the synthesis of compounds **17a** and **17b**

A solution of 2,3-dimethoxybenzoic acid (2.0 g) and a catalytic amount of DMF in thionyl chloride (5 mL) was stirred at 80–90 °C for 3 h. After concentration under reduced pressure, the acid chloride **16** was dissolved in dried CH_2_Cl_2 _and reacted with furan (3 mL) catalyzed by AlCl_3_. The reaction mixture was stirred at 0 °C for 2 h and then warmed to room temperature and stirred for additional 12 h. After the reaction was completed, the reaction was quenched by carefully pouring the mixture into iced water (100 mL) and the resultant compound was collected by filtration. Then, the filtrate was extracted with CH_2_Cl_2_ (3 × 20 mL), and dried with anhydrous Na_2_SO_4_. The solvent was removed under reduced pressure to yield a brown oil that was purified by silica gel column chromatography (petroleum ether/EtOAc 80:20, v/v) to give *(2,3-dimethoxyphenyl)(furan-2-yl)methanone* (**17a**) as light yellow oil (1.78 g, 70% yield). Molecular formula (MW): C_13_H_12_O_4_ (232 g/mol); ^1^H-NMR (CDCl_3_) δ 7.67 (d, 1H, *J* = 1.0 Hz, 5’-H), 7.15 (dd, 1H, *J* = 8.0 Hz, *J* = 7.6 Hz, Ph-5-H), 7.08 (dd, 1H, *J* = 8.4 Hz, *J* =1.6 Hz, Ph-4-H), 7.06 (d, *J* = 3.6 Hz, 1H, 3’-H), 7.02 (dd, 1H, *J* = 7.6 Hz, *J* = 1.6 Hz, Ph-6-H), 6.55 (dd, *J* = 3.6 Hz, *J* = 2.0 Hz, 1H, 4’-H), 3.92 (s, 3H, OCH_3_), 3.83 (s, 3H, OCH_3_); ^13^C-NMR (CDCl_3_) δ 56.0, 62.1, 112.3, 114.7, 120.5, 121.1, 123.9, 133.4, 147.1, 147.5, 152.7, 152.8, 182.8; ESI-MS (%): *m/z* = 233 (100) [M+H]^+^ 255 (100) ([M+Na].

*(2,6-Dimethoxyphenyl)(furan-2-yl)methanone* (**17b**). Mol. formula (MW): C_13_H_12_O_4_ (232 g/mol); light yellow solid; Yield: 85%; mp: 64–66 °C; ^1^H-NMR (CDCl_3_) δ 7.87 (d, 1H, *J* = 1.0 Hz, 5’-H) 7.42 (dd, 1H, *J* = 8.0 Hz, *J* = 8.0 Hz, Ph-4-H), 7.08 (d, 1H, *J* = 8.0 Hz, *J* = 1.6 Hz, Ph-3-H), 7.06 (d, *J* = 3.6 Hz, 1H, 3’-H), 7.02 (dd, 1H, *J* = 8.0 Hz, *J* = 1.2 Hz, Ph-5-H), 6.55 (dd, *J* = 3.6 Hz, *J* = 1.0 Hz, 1H, 4’-H), 3.91 (s, 3H, OCH_3_), 3.83 (s, 3H, OCH_3_); ^13^C-NMR (CDCl_3_) δ 56.0, 62.5, 112.3, 114.7, 120.5, 121.1, 123.9, 133.3, 147.1, 147.5, 152.7, 152.8, 182.8; ESI-MS (%): *m/z* = 233 (100) [M+H]^+^ 255 (100) [M+Na].

#### 3.2.5. General procedure for the synthesis of compounds **21a–21d**

Br_2_ (2 mL) was slowly added to a solution of 4-methoxybenzoic acid (5.0 g) in CCl_4_ (20 mL). The reaction mixture was stirred at 40–45 °C for 24 h. After concentration under reduced pressure, the residue was re-crystallized from boiling water to give 3-bromo-4-methoxybenzoic acid (**19**) as white needles. To chlorinate the methoxybenzoic acid, we used SO_2_Cl_2_. A solution of **19** (2.0 g) and a catalytic amount of DMF in thionyl chloride (5 mL) was stirred at 80–90 °C for 3 h. After concentration under reduced pressure, the acid chloride **20** was dissolved in dried CH_2_Cl_2_ and reacted with furan (3 mL) catalyzed by AlCl_3_. The reaction mixture was stirred at 0 °C for 2 h and then warmed to room temperature and stirred for an additional 12 h. After the reaction was completed, it was quenched by carefully pouring the mixture into iced water (100 mL) and the resultant compound was collected by filtration. The filtrate was then extracted with CH_2_Cl_2_ (3 × 20 mL) and dried with anhydrous Na_2_SO_4_. The solvent was removed under reduced pressure to yield a brown oil that was purified by silica gel column chromatography (petroleum ether/EtOAc 80:20, v/v) to give compound **21a** as a light yellow solid (1.85 g, 76% yield). 

*(3-Bromo-4-methoxyphenyl)(furan-2-yl)methanone*(**21a**). Mol. formula (MW): C_12_H_9_BrO_3_ (281 g/mol); mp: 112–114 °C; ^1^H-NMR (CDCl_3_) δ 8.30 (d, 1H, *J* = 2.0 Hz, 5’-H), 8.06 (dd, 1H, *J* = 8.0 Hz, *J* = 2.0 Hz, Ph-6-H), 7.93 (s, 1H, Ph-2-H), 7.30 (d, 1H, *J* = 3.6 Hz, 3’-H), 7.01 (d, 1H, *J* = 8.0 Hz, Ph-5-H), 6.63 (dd, *J* =3.6 Hz, *J* = 2.0 Hz, 1H, 4’-H), 4.00 (s, 3H, OCH_3_); ^13^C-NMR (CDCl_3_) δ 56.5, 111.1, 112.3, 113.7, 120.1, 130.7, 131.7, 134.9, 146.9, 152.3, 159.4, 179.7; ESI-MS (%): *m/z* = 281(100) 283 (98.7) [M+H]^+^ 303 (100) 305 (98.7) [M+Na].

*(2,6-Dibromo-3,4,5-trimethoxyphenyl)(furan-2-yl)methanone* (**21b**). Mol. formula (MW): C_14_H_12_Br_2_O_5_ (420 g/mol); light yellow solid; Yield: 85%; mp: 96–98 °C; ^1^H-NMR (CDCl_3_) δ 7.71 (d, *J* = 1.0 Hz, 1H, 5’-H), 7.13 (d, *J* = 3.6 Hz, 1H, 3’-H), 6.61 (dd, *J* = 3.6 Hz, *J* = 1.6 Hz, 1H, 4’-H), 4.01 (s, 9H, OCH_3_); ^13^C-NMR (CDCl_3_) δ 61.2, 61.4, 61.4, 110.1, 112.9, 121.2, 136.0, 148.3, 148.6, 151.0, 151.1, 180.5; ESI-MS (%): *m/z* = 419 (51) 421 (100) 423 (50) [M+H]^+^ 441 (51) 443 (100) 445 (50) [M+Na] 457 (51) 459 (100) 461 (50) [M+K].

*(2-Chloro-3,4,5-trimethoxyphenyl)(furan-2-yl)methanone*(**21c**). Mol. formula (MW): C_14_H_13_ClO_5_ (297 g/mol); light yellow solid; Yield: 82%; mp: 80–82 °C; ^1^H-NMR (CDCl_3_) δ 7.74 (s, 1H, 5’-H), 7.12 (d, *J* = 3.6 Hz, 1H, 3’-H), 6.80 (s, 1H, Ph-6-H), 6.60 (dd, *J* = 3.6 Hz, *J* = 2.0 Hz, 1H, 4’-H), 3.97 (s, 9H, OCH_3_); ^13^C-NMR (CDCl_3_) δ 56.3, 61.3, 61.3, 107.0, 107.8, 112.7, 121.4, 132.9, 145.2, 147.9, 150.3, 152.1, 153.0, 181.5; ESI-MS (%): *m/z* = 297 (100) 299 (34) [M+H]^+^ 319 (100) 321 (34) [M+Na] 335 (100) 337 (34) [M+K]; HRMS (ESI): Calcd. for [M+Na]: 319.0344; Found: 319.0343.

*(4,5-Dibromo-2-hydroxy-3-methoxyphenyl)(furan-2-yl)methanone* (**21d**). Mol. formula (MW): C_12_H_8_Br_2_O_4_ (376 g/mol); light yellow solid; Yield: 80%; mp: 102–104 °C; ^1^H-NMR (DMSO-d6) δ 9.86 (br s, 1H, OH), 8.07 (d, 1H, *J* = 1.2 Hz, 5’-H), 7.44 (s, 1H, Ph-6-H), 7.20 (d, *J* = 3.6 Hz, 1H, 3’-H), 6.74 (dd, *J* = 3.6 Hz, *J* = 2.0 Hz, 1H, 4’-H), 3.89 (s, 3H, OCH_3_); ^13^C-NMR (DMSO-d6) δ 57.0, 111.4, 113.6, 113.6, 117.3, 121.6, 129.6, 144.8, 148.6, 149.5, 151.6, 180.6; ESI-MS (%): *m/z* = 375 (51) 377 (100) 379 (50) [M+H]^+^ 397 (51) 399 (100) 401 (50) [M+Na]; HRMS (ESI): Calcd. for [M+Na]: 396.8682; Found: 396.8683.

#### 3.2.6. General procedure for the synthesis of compounds **4a–4d**, **8a–8c**, **14**, **18a–18b** and **22a–22d**

10% (equiv.) BBr_3_ (2 mL) was added to a solution of compound **3a** (0.5 g) in CH_2_Cl_2_ (20 mL). The reaction mixture was stirred at −78 °C for 30 min and then warmed to room temperature and stirred for additional 3.5 h. After the reaction was completed, it was quenched by carefully pouring the mixture into iced water (100 mL), extraction of the aqueous layer three times with EtOAc, washing with 5% NaHSO_3_ (40 mL) and water (100 mL), and drying with anhydrous Na_2_SO_4_. The solvent was removed under reduced pressure to yield a light red solid compound **4a** (0.403 g, 91.7% yield). 

*Synthesis of (3,4-Dihydroxyphenyl)(furan-2-yl)methanone* (**4a**).Mol. formula (MW): C_11_H_8_O_4_ (204 g/mol); mp: 132–134 °C; ^1^H-NMR (CDCl_3_) δ 7.58 (d, *J* = 8.0 Hz, 1H, Ph-5-H), 7.53 (s, 1H, 5’-H), 7.40 (s, 1H, Ph-2-H), 7.10 (d, *J* = 3.2 Hz, 1H, 3’-H), 6.81 (d, *J* = 8.0 Hz, 1H, Ph-6-H), 6.46 (s, 1H, 4’-H); ^13^C-NMR (CDCl_3_) δ 111.8, 114.9, 116.5, 119.7, 124.0, 129.8, 146.6, 148.9, 152.3, 153.0, 180.1; ESI-MS (%): *m/z* = 205 (100) [M+H]^+^; HRMS (ESI): Calcd. for [M+Na]: 227.0315; Found: 227.0310.

*Furan-2-yl(2,3,4-trihydroxyphenyl)methanone* (**4b**). Mol. formula (MW): C_11_H_8_O_5_ (220 g/mol); light red solid; Yield: 95%; mp: 168–170 °C; ^1^H-NMR (DMSO-d6) δ 7.73 (d, *J* = 1.6 Hz, 1H, 5’-H), 7.64 (d, *J* = 9.2 Hz, 1H, Ph-6-H), 7.44 (d, *J* = 3.6 Hz, 1H, 3’-H), 6.78 (dd, *J* = 3.6 Hz, *J* = 1.6 Hz, 1H, 4’-H), 6.53 (d, *J* = 9.2 Hz, 1H, Ph-5-H); ^13^C-NMR (DMSO-d6) δ 108.6, 113.1, 113.2, 120.9, 123.7, 148.5, 148.7, 151.8, 152.9, 153.3, 183.5; ESI-MS (%): *m/z* = 219 (100) [M−H]^−^; HRMS (ESI): Calcd. for [M+Na]: 243.0264; Found: 243.0261.

*(5-Bromo-2-hydroxyphenyl)(furan-2-yl)methanone* (**4c**). Mol. formula (MW): C_11_H_7_BrO_3_ (267 g/mol); light red solid; Yield: 94%; mp: 88–90 °C; ^1^H-NMR (DMSO-d6) δ 10.50 (br s, 1H, OH), 8.08 (d, *J* = 1.0 Hz, 1H, 5’-H), 7.58 (d, *J* = 2.4 Hz, 1H, Ph-6-H), 7.56 (d, *J* = 8.4 Hz, 1H, Ph-4-H), 7.26 (d, *J* = 3.6 Hz, 1H, 3’-H), 6.95 (d, *J* = 8.4 Hz, 1H, Ph-3-H), 6.76 (dd, *J* = 3.6 Hz, *J* = 2.0 Hz, 1H, 4’-H); ^13^C-NMR (CDCl_3_) δ 110.3, 113.4, 119.4, 121.7, 127.5, 131.8, 135.5, 149.1, 152.2, 156.0, 181.7; ESI-MS (%): *m/z* = 265 (100) 267 (98.6) [M−H]^−^; HRMS (ESI): Calcd. for [M+Na]: 288.9471; Found: 288.9473.

*Furan-2-yl(4-hydroxyphenyl)methanone* (**4d**). Mol. formula (MW): C_11_H_8_O_3_ (188 g/mol); light red solid; Yield: 95%; mp: 168–170 °C; ^1^H-NMR (DMSO-d6) δ 10.44 (br s, 1H, OH), 8.07 (d, *J* = 1 Hz, 1H, 5’-H), 7.89 (s, 1H, Ph-2-H), 7.87 (s, 1H, Ph-6-H), 7.33 (d, *J* = 3.6 Hz, 1H, 3’-H), 6.93 (s, 1H, Ph-5-H), 6.91 (s, 1H, Ph-3-H), 6.76 (dd, *J* = 3.6 Hz, *J* = 2.4 Hz, 1H, 4’-H); ^13^C-NMR (DMSO-d6) δ 112.9, 115.8, 115.8, 120.3, 128.3, 132.1, 132.1, 148.1, 152.2, 162.4, 180.4; ESI-MS (%): *m/z* = 187 (100) [M−H]^−^; HRMS (ESI): Calcd. for [M+Na]: 211.0366; Found: 211.0368.

*(5-Bromofuran-2-yl)(2,3,4-trihydroxyphenyl)methanone* (**8a**). Mol. formula (MW): C_11_H_7_BrO_5_ (299 g/mol); light red solid; Yield: 95%; mp: 132–134 °C; ^1^H-NMR (DMSO-d6) δ 7.48 (d, *J* = 8.8 Hz, 1H, Ph-6-H), 7.43 (d, *J* = 3.6 Hz, 1H, 3’-H), 6.94 (d, *J* = 3.6 Hz, 1H, 4’-H), 6.50 (d, *J* = 8.8 Hz, 1H, Ph-5-H); ^13^C-NMR (DMSO-d6) δ 182.0, 158.7, 153.3, 153.1, 147.3, 127.2, 123.3, 120.5, 114.8, 112.6, 108.7; ESI-MS (%): *m/z* = 297 (100) 299 (99) [M−H]^−^; HRMS (ESI): Calcd. for [M+Na]: 320.9369; Found: 320.9374.

*(5-Bromo-2-hydroxyphenyl)(5-bromofuran-2-yl)methanone* (**8b**). Mol. formula (MW): C_11_H_6_Br_2_O_3_ (346 g/mol); light red solid; Yield: 95%; mp: 82–84 °C; ^1^H-NMR (DMSO-d6) δ 10.46 (br s, 1H, OH), 7.57 (d, *J* = 8.0 Hz, 1H, Ph-4-H), 7.54 (s, *J* = 8.0 Hz, 1H, Ph-6-H), 7.25 (d, *J* = 3.6 Hz, 1H, 3’-H), 6.95 (d, *J* = 8.0 Hz, 1H, Ph-3-H), 6.90 (d, *J* = 3.6 Hz, 1H, 4’-H); ^13^C-NMR (DMSO-d6) δ 110.3, 115.6, 119.4, 123.8, 127.1, 130.0, 131.9, 135.6, 153.4, 155.7, 180.3; ESI-MS (%): *m/z* = 343 (51) 345 (100) 347 (50) [M−H]^−^; HRMS (ESI): Calcd. for [M+H]^+^: 344.8736; Found: 344.8742.

*(5-Bromofuran-2-yl)(3,4-dihydroxyphenyl)methanone* (**8c**). Mol. formula (MW): C_11_H_7_BrO_4_ (283 g/mol); light red solid; Yield: 95%; mp: 138–140 °C; ^1^H-NMR (CDCl_3_) δ 7.54 (d, *J* = 2.0 Hz, 1H, Ph-2-H), 7.48 (dd, *J* = 8.4 Hz, *J* = 1.6 Hz, 1H, Ph-6-H), 7.12 (d, *J* = 3.6 Hz, 1H, 3’-H), 6.94 (d, *J* = 8.4 Hz, 1H, Ph-5-H), 6.49 (d, *J* = 3.6 Hz, 1H, 4’-H); ^13^C-NMR (CDCl_3_) δ 118.8, 120.0, 121.3, 126.2, 127.6, 132.7, 133.0, 150.0, 155.4, 158.9, 184.4; ESI-MS (%): *m/z* = 283 (100) 285 (98.8) [M+H]^+^; HRMS (ESI): Calcd. for [M+Na]: 304.9420; Found: 304.9420.

*(4,5-Dibromofuran-2-yl)(3,4-dihydroxyphenyl)methanone* (**14**). Mol. formula (MW): C_11_H_6_Br_2_O_4_ (362 g/mol); light red solid; Yield: 91%; mp: 184–186 °C; ^1^H-NMR (CDCl_3_) δ 7.53 (s, 1H, Ph-2-H), 7.48 (d, *J* = 8.0 Hz, 1H, Ph-6-H), 7.18 (s, 1H, 3’-H), 6.95 (d, *J* = 8.0 Hz, 1H, Ph-5-H); ^13^C-NMR (CDCl_3_) δ 104.0, 115.2, 116.5, 121.3, 122.8, 127.7, 128.6, 145.1, 150.9, 158.4, 179.0; ESI-MS (%): *m/z* = 359 (51) 361 (100) 363 (50) [M−H]^−^; HRMS (ESI): Calcd. for [M+Na]: 382.8531; Found: 382.8533.

*(2,3-Dihydroxyphenyl)(furan-2-yl)methanone* (**18a**). Mol. formula (MW): C_11_H_8_O_4_ (204 g/mol); light red solid; Yield: 95%; mp: 64–66 °C; ^1^H-NMR (DMSO-d6) δ 8.06 (d, 1H, *J* = 1.0 Hz, 5’-H), 7.30 (d, *J* = 3.6 Hz, 1H, 3’-H), 7.10 (dd, 1H, *J* = 8.0 Hz, *J* = 1.6 Hz, Ph-6-H), 7.06 (dd, 1H, *J* = 8.4 Hz, *J* = 2.0 Hz, Ph-4-H), 6.91 (dd, 1H, *J* = 8.4 Hz, *J* = 8.0 Hz, Ph-5-H), 6.72 (dd, *J* = 3.6 Hz, *J* = 2.0 Hz, 1H, 4’-H); ^13^C-NMR (DMSO-d6) δ 109.4, 113.1, 113.6, 120.1, 121.5, 130.0, 144.3, 146.8, 149.4, 151.8, 180.9; ESI-MS (%): *m/z* = 203 (100) [M−H]^−^; HRMS (ESI): Calcd. for [M+Na]: 227.0315; Found: 227.0313.

*(2,6-Dihydroxyphenyl)(furan-2-yl)methanone* (**18b**). Mol. formula (MW): C_11_H_8_O_4_ (204 g/mol); light red solid; Yield: 95%; mp: 66–68 °C; ^1^H-NMR (DMSO-d6) δ 8.08 (d, 1H, *J* = 1.0 Hz, 5’-H), 7.30 (d, *J* = 3.6 Hz, 1H, 3’-H), 7.17 (dd, 1H, *J* = 8.0 Hz, *J* = 1.6 Hz, Ph-3-H), 7.04 (dd, 1H, *J* = 8.4 Hz, *J* = 2.0 Hz, Ph-5-H), 6.81 (dd, 1H, *J* = 8.4 Hz, *J* = 8.0 Hz, Ph-4-H), 6.76 (dd, *J* = 3.6 Hz, *J* = 2.0 Hz, 1H, 4’-H); ^13^C-NMR (DMSO-d6) δ 113.2, 119.4, 119.6, 120.5, 121.6, 123.9, 146.5, 147.4, 148.9, 152.2, 184.0; ESI-MS (%): *m/z* = 203 (100) [M−H]^−^; HRMS (ESI): Calcd. for [M+Na]: 227.0315; Found: 227.0310.

*(3-Bromo-4-hydroxyphenyl)(furan-2-yl)methanone* (**22a**). Mol. formula (MW): C_11_H_7_BrO_3_ (267 g/mol); light red solid; Yield: 90%; mp: 100–102 °C; ^1^H-NMR (DMSO-d6) δ 11.32 (br s, 1H, OH), 8.10 (d, 1H, *J* = 2.8 Hz, Ph-2-H), 8.09 (d, 1H, *J* = 2.0 Hz, 5’-H), 7.90 (dd, 1H, *J* = 8.8 Hz, *J* = 2.4 Hz, Ph-6-H), 7.40 (d, *J* = 3.6 Hz, 1H, 3’-H), 7.12 (d, 1H, *J* = 8.8 Hz, Ph-5-H), 6.79 (dd, *J* = 3.6 Hz, *J* = 2.0 Hz, 1H, 4’-H); ^13^C-NMR (DMSO-d6) δ 110.0, 113.1, 116.5, 120.9, 129.6, 131.1, 134.7, 148.6, 151.9, 158.9, 179.2; ESI-MS (%): *m/z* = 265 (100) 267 (98.6) [M−H]^−^; HRMS(ESI): Calcd. for [M+Na]: 288.9472; Found: 288.9470.

*(2,6-Dibromo-3,4,5-trihydroxyphenyl)(furan-2-yl)methanone* (**22b**). Mol. formula (MW): C_11_H_6_Br_2_O_5_ (378 g/mol); light red solid; Yield: 93%; mp: 148–150 °C; ^1^H-NMR (DMSO-d6) δ 9.82 (br s, 2H, OH), 9.58 (br s, 1H, OH), 8.07 (d, *J* = 1.6 Hz, 1H, 5’-H), 7.13 (d, *J* = 3.6 Hz, 1H, 3’-H), 6.74 (dd, *J* = 3.6 Hz, *J* = 1.6 Hz, 1H, 4’-H); ^13^C-NMR (DMSO-d6) δ 99.1, 113.6, 113.7, 121.9, 130.9, 136.9, 143.8, 143.8, 149.7, 151.3, 181.4; ESI-MS (%): *m/z* = 375 (51) 377 (100) 379 (50) [M−H]^−^; HRMS (ESI): Calcd. for [M+H]^+^: 378.8635; Found: 378.8637.

*(2-Chloro-3,4,5-trihydroxyphenyl)(furan-2-yl)methanone* (**22c**). Mol. formula (MW): C_11_H_7_ClO_5_ (255 g/mol); light red solid; Yield: 94%; mp: 150–152 °C; ^1^H-NMR (DMSO-d6) δ 9.65 (br s, 3H, OH), 9.42 (br s, 3H, OH), 8.07 (s, 1H, 5’-H), 7.14 (d, *J* = 3.6 Hz, 1H, 3’-H), 6.74 (dd, *J* = 3.6 Hz, *J* = 1.6 Hz, 1H, 4’-H), 6.25 (s, 1H, Ph-6-H); ^13^C-NMR (DMSO-d6) δ 108.1, 113.3, 121.8, 127.8, 137.4, 143.5, 144.5, 146.1, 149.1, 152.2, 181.4; ESI-MS (%): *m/z* = 253 (100) 255 (33.7) [M−H]^−^; HRMS (ESI): Calcd. for [M+Na]: 276.9874; Found: 276.9876.

*(4,5-Dibromo-2,3-dihydroxyphenyl)(furan-2-yl)methanone* (**22d**). Mol. formula (MW): C_11_H_6_Br_2_O_4_ (362 g/mol); light red solid; Yield: 95%; mp: 138–140 °C; ^1^H-NMR (DMSO-d6) δ 9.66 (br s, 1H, OH), 9.37 (br s, 1H, OH), 8.07 (s, 1H, 5’-H), 7.40 (s, 1H, Ph-6-H), 7.14 (d, *J* = 3.6 Hz, 1H, 3’-H), 6.74 (dd, *J* = 3.6 Hz, *J* = 2.0 Hz, 1H, 4’-H); ^13^C-NMR (DMSO-d6) δ 108.1, 109.3, 113.3, 120.0, 121.8, 127.8, 143.5, 144.5, 149.1, 152.2, 181.4; ESI-MS (%): *m/z* = 359 (51) 361 (100) 363 (50) [M−H]^−^; HRMS (ESI): Calcd. for [M+Na]: 382.8526; Found: 382.8528.

## 4. Biological Evaluation

### 4.1. PTK Inhibitory Activity

The activities of PTKs were tested using ELISA. The tyrosine kinase was extracted from brain tissue of rat, and microtiter plates were coated using poly-Glu-Tyr (PGT) as substrates. If the tyrosine residues of PGT were phosphorylated by PTKs, they bound to phospho-specific monoclonal antibody that was labeled specifically with HRP. The absorbance was measured to reflect the activity of PTK.

### 4.2. Tyrosine Kinase Assay

The phosphorylation assays were performed at 37 °C in a final volume of 40 µL tyrosine kinase. The concentrations of PTKs used to construct calibration curves were as follows: 600, 500, 400, 300, 200 and 100 × 10^−7^ U/mL for PTK. A concentration of 500 × 10^−7^/μL was used for each inhibitor. Phosphorylation reactions were initiated with the addition of 40 mM ATP (10 μL) into each vessel, and the plate was incubated at 37 °C for 30 min. After completion of reaction, liquid was decanted and the vessels were washed four times with Tween-PBS. One hundred microliters of blocking solution was added to the vessels and incubated at 37 °C for 30 min. After washing the plate with Tween-PBS, anti-phosphotyrosine (50 μL) was added to the vessels and incubated at 37 °C for 30 min. The reaction liquid was decanted and the remaining solution was removed by rinsing four times with Tween-PBS. One hundred μL of HRP coloring agent was added and incubated at 37 °C for 15 min. The reaction was terminated by addition of 1 N sulfuric acid (100 μL /well). The absorbance of the reaction was measured at 450 nm on a microplate reader.

## 5. Conclusions

In summary, a number of new furan-2-yl(phenyl) methanone derivatives were synthesized and evaluated for their *in vitro* PTK inhibitory activity. Several new derivatives exhibited promising activity, which in some cases was identical to, or even stronger than, that of genistein, a positive reference compound.

The results justify further studies on these types of derivatives with significant pharmacological (PTK inhibitory) value.
